# Association of nocturnal sleep duration and midday napping with subjective poor hearing among middle-aged and older adults in China

**DOI:** 10.3389/fpubh.2023.1160294

**Published:** 2023-04-11

**Authors:** Xiaorui Cui, Zixuan Lu, Xinyue Guo, Neng Dai, Chen Huang, Yahang Liu, Ruilang Lin, Yongfu Yu, Guoyou Qin, Jiaohua Chen

**Affiliations:** ^1^Department of Biostatistics, The Key Laboratory of Public Health Safety of Ministry of Education, School of Public Health, Fudan University, Shanghai, China; ^2^Department of Health Management, Seventh People's Hospital of Shanghai University of Traditional Chinese Medicine, Shanghai, China; ^3^Department of Cardiology, Zhongshan Hospital, Shanghai Institute of Cardiovascular Diseases, Fudan University, Shanghai, China; ^4^National Clinical Research Center for Interventional Medicine, Shanghai, China; ^5^Shanghai Institute of Infectious Disease and Biosecurity, Shanghai, China

**Keywords:** nocturnal sleep, midday napping, hearing loss, longitudinal survey, joint effect

## Abstract

**Background:**

Hearing loss has occurred as a critical concern for aging and health. However, it remains unknown whether nocturnal sleep and midday napping duration are associated with hearing loss in middle-aged and older adults.

**Methods:**

The study comprised 9,573 adults from China Health and Retirement Longitudinal Study, who have completed the survey for sleep characteristics and subjective functional hearing. We collected self-reported nocturnal sleep duration (<5, 5 to <6, 6 to <7, 7 to <9, ≥9 h/night) and midday napping duration (≤5, 5 to ≤30, and >30 min). The sleep information was classified into different sleep patterns. The primary outcome was self-reported hearing loss events. Multivariate Cox regression models and restricted cubic splines were used to investigate the longitudinal association of sleep characteristics with hearing loss. We applied Cox generalized additive models and bivariate exposure-response surface diagrams to visualize the effects of different sleep patterns on hearing loss.

**Results:**

We confirmed 1,073 cases of hearing loss (55.1% female) during the follow-up. After adjusting for demographic characteristics, lifestyle factors and health condition, nocturnal sleep with < 5 h was positively associated with hearing loss [hazard ratio (HR): 1.45, 95% confidence interval [CI]: 1.20, 1.75]. Individuals with napping for 5 to ≤30 min had a 20% (HR: 0.80, 95%CI: 0.63, 1.00) lower risk of hearing loss compared with those with napping ≤ 5 min. Restrictive cubic splines showed the reverse J-shaped association between nocturnal sleep and hearing loss. Moreover, we found significant joint effects of sleeping < 7 h/night and midday napping ≤ 5 min (HR: 1.27, 95% CI: 1.06, 1.52) on hearing loss. Bivariate exposure-response surface diagrams also reflected the finding that short sleep without napping existed the highest risk of hearing loss. Compared with persistently sleeping moderately (7–9 h/night), those who persistently slept < 7 h/night or shifted from < 7 h/night to moderate or > 9 h/night had higher risks of hearing loss.

**Conclusion:**

Inadequate nocturnal sleep was associated with an elevated risk of poor subjective hearing in middle-aged and older adults, while moderate napping decreased the risk of hearing loss. Keeping sleep stable within recommendation duration may be a useful strategy for preventing poor hearing loss.

## 1. Introduction

According to a nationwide investigation, 58% of adults over 60 had hearing loss (HL) diagnosed (1). HL has occurred as the main contributor of disability-adjusted life-years (DALYs) among the population aged over 50, making it a critical issue for aging and health (2–4). Previous research reported that HL may be related to both physical and mental problems, increasing the risks of dementia, social isolation, cognitive decline, frailty, mortality, etc. in the elder population (5–8). Despite its prevalence and severity, it may be underrecognized due to the misconception that HL is a normal part of aging (9).

Sleep architecture changes with growing age in human beings, which are embodied in shorter sleep duration, sleep fragment and poorer sleep quality (10). It was reported that sleep might affect hearing condition by disrupting fluid absorption and ion homeostasis, destructing hair cells in the cochlea and changing neural input from the central auditory system (11–13). Being considered as a behavior distinct from nocturnal sleep, midday napping is common in both many countries and more prevalent in the old (14–17). Napping has been shown to benefit older adults by improving well-being and cognition (18). Furthermore, sleep at night and midday napping may influence mutually, with nappers reporting more difficulty falling and staying asleep at night (19, 20). Studies have portrayed that different patterns of nocturnal sleep and napping have complex effects on health events. In this context, it is critical to explore their conjoint effect (17, 21, 22).

Change in sleep duration was also highlighted as a predictor of negative health outcomes (23–25). However, it is yet uncertain how the comprehensive sleep patterns, as well as changes in nocturnal sleep duration and midday napping duration, relate to the risk of HL.

Research from China and America have suggested the U-shaped linkage between nocturnal sleep duration and HL that the risk of HL increased with sleeping shorter than 6 or longer than 8 h/night (26, 27). The other work demonstrated different results. Each additional hour of sleep is related to worse high-frequency hearing among participants who slept more than 8 h in an American cohort (28). A Japan study observed that compared with 6 h, prolonged sleep duration was significantly associated with incident HL (29). However, the cross-sectional analysis could not avoid reverse causality and restricted the strength of evidence. Additionally, these articles did not include the measurement of napping and describe sleep patterns comprehensively. Therefore, the longitudinal relationships between sleep characteristics, sleep patterns and HL remain unclear.

This study aimed to estimate the correlation between nocturnal sleep duration, midday napping duration and subsequent HL among a large cohort of Chinese adults and to provide more longitudinal evidence. We also assessed the effect of different sleep patterns as well as changes in nocturnal sleep and napping on HL risk.

## 2. Methods

### 2.1. Study population

The study was conducted based on the China Health and Retirement Longitudinal Study (CHARLS), which is a prospective and representative cohort study enrolling a large population aged ≥ 45 years. The survey compiles extensive health data of community residents through face-to-face personal interviews with the structured questionnaire. Detailed information has been previously published (30). The CHARLS survey project was approved by the Biomedical Ethics Review Committee of Peking University, and informed consent forms were acquired from all participants.

**Figure 1:**
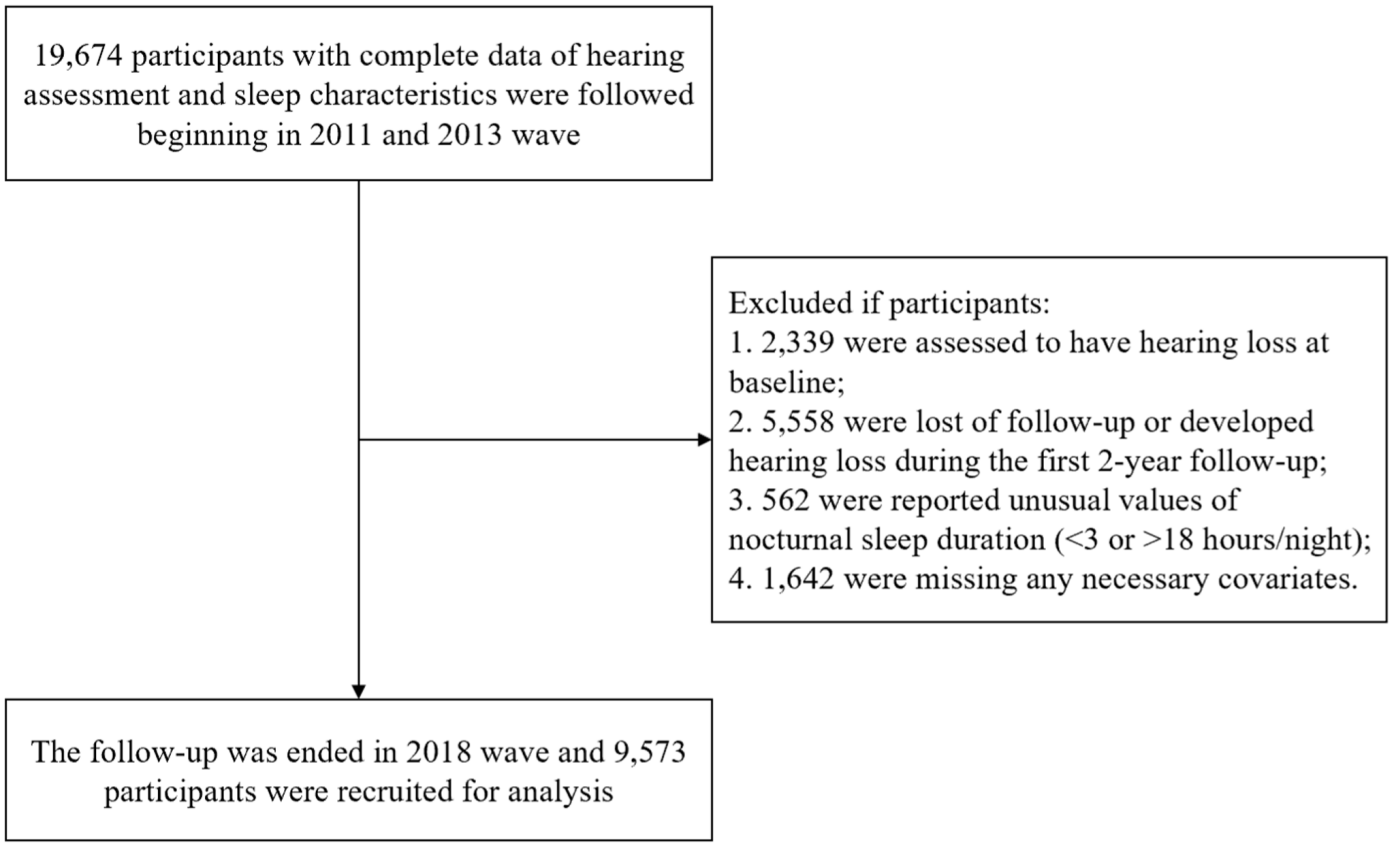
Flow chart of filtration.

A baseline survey was conducted in 2011 and follow-ups were on average every 2–3 years until 2018. In this study, we originally included 19,674 participants who reported sleep characteristics at baseline and the second visit with completed hearing assessments. The procedure of sample selection is displayed in Figure 1.

### 2.2. Sleep characteristic assessment

Nocturnal sleep duration was numerically examined by the subjective answer to the question: “During the past month, how many hours of actual sleep did you get at night?” The answers were then categorized into five groups (<5, 5 to <6, 6 to <7, 7 to <9, ≥9 h/night). According to the recommendation from the National Sleep Foundation, the 7 to <9 h group was treated as the reference group (31).

Midday napping duration was collected by asking “During the past month, how long did you take a nap after lunch?.” Based on previous studies, midday napping with ≤ 5 min may have limited recuperative power compared to non-nappers, and therefore the respondents were categorized into three groups (≤5, 5 to ≤30, and >30 min) through treated naps ≤ 5 min as no napping group (31, 32). The group of ≤ 5 min was used as the reference.

We defined total sleep duration as nocturnal sleep duration plus midday napping duration.

### 2.3. Definition of hearing loss

Respondents were required to answer a single-item scale: “Would you say your hearing is excellent, very good, good, fair, or poor?” In order to focus on more clinically meaningful outcomes including moderate to severe hearing loss and minimize potential misclassification, we defined the answers of “excellent,” “very good,” “good” and “fair” as “not having HL,” while “poor” was defined as “having HL” (33–36). In our study, HL refers to self-perceived functional hearing more than ability disorder (37).

### 2.4. Covariates

We collected potential confounders including demographic variables, lifestyle risk factors and health condition. Age, gender, area of residence (urban or rural), education (below primary school, middle school, high school or above) and marital status (married or not). Body mass index (BMI, kg/m^2^) was calculated as weight divided by height squared. We measured lifestyle with smoking (ever or never smokers) and drinking status (current or non-drinkers). Social participation was measured by a multiple-choice question “Have you done any of these activities in the last month?” The answers include 11 activities and the total numbers were divided into three groups: None, 1 type and ≥ 2 types (38). Health condition was assessed by self-reported hypertension, diabetes, stroke, heart disease, psychiatric disease, memory-related disease, dyslipidemia, cancer and chronic lung disease by asking the question such as “Have you been diagnosed with a condition by a doctor?” with standardized questionnaire and computer-assisted personal interview technology. Furthermore, the answers of participants were double-checked with the question “Is the disease record from the last survey correct?” to minimize the potential risk of recall bias. The question “How often do you feel the quality of your sleep is bad during sleep last week?” was used to assess sleep quality and responses were categorized into “good” (<1 day), “fair” (1–2 or 3–4 days) and “poor” (5–7 days) (39).

### 2.5. Statistical analysis

Baseline characteristics were provided as median (interquartile range) for continuous variables and frequencies (%) for categorical ones. The Wilcoxon signed-rank tests were used to analyze differences in characteristics, while the *χ*^2^ tests and the Wilcoxon rank tests were for categorical ones.

We used multiple linear regression to calculate the variance inflation factor (VIF) to check the collinearity of variables and assessed the proportional hazards (PH) assumption by examining log (−log) survival plots. The separate and joint associations between sleep characteristics and HL were determined by Cox proportional hazards models and calculated as hazard ratios (HRs) and 95% confidence intervals (CIs). Latent confounders were chosen based on a directed acyclic graph (DAG; Supplementary Figure S1). We first estimated the crude HRs in unadjusted models, and then adjusted for gender, age, marital status, education level, area of residence, smoking status, drinking status, BMI, social participation, baseline chronic condition status and sleep quality. For the analysis of joint effect, we combined extreme duration of nocturnal sleep groups to conserve statistical power, with a sleep pattern of moderate nocturnal sleep without napping set as the reference group. Furthermore, stratified analysis by different categories of nocturnal sleep and napping duration was used to investigate the association closely.

As the known nonlinear relationships between sleep duration and various health outcomes, we further detect the nonlinear patterns of association mentioned above. Restricted cubic splines (RCS) with nocturnal sleep duration in hours and napping duration in minutes as continuous variables and duration of 7 h and 0 min as the reference point, respectively. To model the underlying connection, three knots were placed at the 5th, 50th, and 95th percentiles. A *value of p* of non-linear < 0.05 suggested a non-linear association between the exposure and HL.

We further explored the relationship between changes in duration of nocturnal sleep, midday napping and the occurrence of HL. Changes in nocturnal sleep duration between baseline and subsequent visits were classified into 9 patterns by combining 3 groups (<7, 7 to <9, ≥9 h/night). Similarly, three categories of midday napping duration (≤5, 5 to ≤30, and > 30 min) were used to create 9 subgroups of changes. The individuals who persistently slept 7 to <9 h/night or took ≤5-min naps were treated as the reference groups, respectively.

The Cox generalized additive model (CGAM) was applied to estimate nonlinear or nonparametric relationships between nocturnal sleep, napping and the risks of HL more flexibly by smoothers (40). The bivariate model was built as followed, where h(t,X) denoted the hazard function at t times under the risk factors X, $h_{0}\left(t\right)$ denoted the baseline hazard function at t times with all independent variables were 0, s(nocturnal sleep duration,napping duration) was the bivariate smooth functional relationships.

Other confounders were directly included in the model. The degree of freedom of the smoothing number was set to 6 for s(nocturnal sleep duration,napping duration).


$$\begin{array}{c} \mathrm{Ln}\frac{h\left(t,X\right)}{h_{0}\left(t\right)}=s\left(\mathrm{nocturnal sleep duration,napping duration}\right)\\ +\beta_{1}\mathrm{Gender}+\beta_{2}\mathrm{Age}+\beta_{3}\mathrm{Area}+\beta_{4}\mathrm{Marriage}\\ +\beta_{5}\mathrm{BMI}+\beta_{6}\mathrm{Education}+\beta_{7}\mathrm{Smoking}\\ +\beta_{8}\mathrm{Drinking}+\beta_{9}\mathrm{Activity}+\beta_{10}\mathrm{Hp}+\beta_{11}\mathrm{DM}\\ +\beta_{12}\mathrm{ICVD}+\beta_{13}\mathrm{CVD}+\beta_{14}\mathrm{CVD}+\beta_{15}\mathrm{Psychiatric}\\ +\beta_{16}\mathrm{Memory}+\beta_{17}\mathrm{Dyslipid}+\beta_{18}\mathrm{Cancer}+\beta_{19}\mathrm{Lung}. \end{array}$$

The results were visualized by plotting bivariate exposure-response surface diagrams.

In sensitivity analysis, we re-categorized sleep duration as short (<7 h/day), moderate (7 to <9 h/day) and long (≥ 9 h/day) and re-assigned the reference group as 7-8 h to evaluate the robustness of results. Given that obstructive sleep apnea-hypopnea (OSAH) and snoring may be important confounders (41, 42) and their strong association with obesity and sleep quality shown previously (43, 44), we took BMI as a surrogate for OSAH and snoring and excluded obese participants (BMI ≥ 30 kg/m^2^) to limit their effects. Multiple imputations were applied to handle missing data in Cox regression analysis assuming missing at random. Ten imputed datasets were created and the statistical estimates were pooled to compare results between imputed and complete datasets.

SAS version 9.4 software (SAS Institute, Cary, NC, United States) and R version 4.1.3 (R Foundation for Statistical Computing) were used to analyze the data. All statistical analyses were the two-sided test, and *p* < 0.05 was considered statistically significant.

## 3. Results

### 3.1. General characteristics

Our study enrolled a total of 9,573 participants, and the median age was 56.0 years and 47.6% were men. The median (interquartile range [IQR]) of self-reported nocturnal sleep duration was 7.0 (6.0, 8.0) h and midday napping duration was 1.0 (0.0, 60.0) min at baseline.

Table 1 displays the baseline characteristics of subjects. Compared to the individuals without HL, those who developed HL were more likely to be older, rural residents and highly educated, and less likely to be overweight, married, current drinking and active in social participation (*p* < 0.05). Individuals who developed HL reported higher percentages of hypertension, heart disease, chronic lung disease and bad sleep quality (*p* < 0.05).

**Table 1:** Characteristics of studied population at baseline with or without hearing loss.

Variables	No hearing loss	Hearing loss	*p* value
	*N* = 8,500	*N* = 1,073	
Male, *n* (%)	4,076 (48.0)	482 (44.9)	0.061
Age, *n* (%)			<0.001
<50	2,433 (28.6)	178 (16.6)	
<60	3,161 (37.2)	322 (30.0)	
<70	2,185 (25.7)	384 (35.8)	
≥70	721 (8.5)	189 (17.6)	
BMI category, *n* (%)			<0.001
<18.5	376 (4.4)	91 (8.5)	
<24	4,377 (51.5)	569 (53.0)	
<28	2,663 (31.3)	305 (28.4)	
≥28	1,084 (12.8)	108 (10.1)	
Rural residents, *n* (%)	5,166 (60.8)	750 (69.9)	<0.001
Married, *n* (%)	7,314 (86.1)	882 (82.2)	<0.001
Education level, *n* (%)			<0.001
Below primary school	1,137 (13.4)	61 (5.7)	
Primary school	2063 (24.3)	192 (17.9)	
Middle school	2037 (24.0)	249 (23.2)	
High school or above	3,263 (38.4)	571 (53.2)	
Ever smoking, *n* (%)	3,325 (39.1)	403 (37.6)	0.324
Current drinking, *n* (%)	3,009 (35.4)	334 (31.1)	0.006
Social activity, *n* (%)			<0.001
0	3,856 (45.4)	566 (52.8)	
1	3,828 (45.0)	452 (42.1)	
2 or more	816 (9.6)	55 (5.1)	
Hypertension, *n* (%)	1776 (20.9)	268 (25.0)	0.002
Diabetes, *n* (%)	464 (5.5)	60 (5.6)	0.857
Stroke, *n* (%)	125 (1.5)	19 (1.8)	0.447
Heart disease, *n* (%)	765 (9.0)	139 (13.0)	<0.001
Psychiatric disease, *n* (%)	80 (0.9)	12 (1.1)	0.575
Memory-related disease, *n* (%)	68 (0.8)	7 (0.7)	0.605
Dyslipidemia, *n* (%)	759 (8.9)	89 (8.3)	0.490
Cancer, *n* (%)	65 (0.8)	11 (1.0)	0.365
Chronic lung disease, *n* (%)	642 (7.6)	144 (13.4)	<0.001
Sleep quality, *n* (%)			<0.001
Good	4,640 (54.6)	475 (44.3)	
Fair	2,550 (30.0)	388 (36.2)	
Bad	1,310 (15.4)	210 (19.6)	
Nocturnal sleep duration (hours)	7 (6, 8)	6 (5, 8)	<0.001
<5	892	198	<0.001
5 to <6	1,083	147	
6 to <7	1950	239	
7 to <9	3,891	404	
≥9	684	85	
Midday napping duration (minutes)	2 (0, 60)	1 (0, 60)	0.062
≤5	4,484	606	0.019
5 to ≤30	838	82	
>30	3,178	385	

Data are expressed as median (interquartile range, 25–75%) for continuous variables and proportions for categorical variables. BMI, body mass index.

Baseline characteristics grouped by nocturnal sleep, napping and total sleep duration were displayed in Supplementary Table S1–S3. The characteristics of the subjects included or excluded from the main analyses due to missing data are described in Supplementary Table S4.

### 3.2. The separate and joint association of nocturnal sleep and midday napping duration on hearing loss at baseline

Table 2 summarizes the HR and 95% CIs for the risk of HL across the categories of nocturnal sleep duration and midday napping time.

**Table 2:** Separate and joint effects of sleep characteristics on hearing loss.

Variables	Case (%)	Crude incidence (1,000 person-year)	Crude HR (95%CI)	Adjusted HR^a^ (95%CI)
*Nocturnal sleep duration (hours)*				
<5	198 (18.2)	29.22	1.99 (1.68, 2.36)	1.45 (1.20, 1.75)
5 to <6	147 (12.0)	18.83	1.29 (1.07, 1.56)	1.04 (0.86, 1.28)
6 to <7	239 (10.9)	17.20	1.19 (1.01, 1.39)	1.12 (0.96, 1.32)
7 to <9	404 (9.4)	14.75	1 (ref)	1 (ref)
≥9	85 (11.1)	17.26	1.17 (0.92, 1.47)	1.07 (0.84, 1.35)
*Midday napping duration (minutes)*				
≤5	606 (11.9)	18.64	1 (ref)	1 (ref)
5 to ≤30	82 (8.9)	14.50	0.86 (0.60, 0.95)	0.80 (0.63, 1.00)
>30	385 (10.8)	17.08	0.95 (0.84, 1.09)	0.97 (0.82, 1.11)
*Joint effect*				
*p* for interaction				0.813
*Nocturnal sleep duration (hours)*	*Midday napping duration (minutes)*	Case (%)	Crude incidence (1,000 person-year)	Crude HR (95%CI)	Adjusted HR^a^ (95%CI)
<7	≤5	348 (14.0)	22.11	1.54 (1.29, 1.83)	1.27 (1.06, 1.52)
7 to <9	≤5	204 (9.3)	14.69	1 (ref)	1 (ref)
≥9	≤5	54 (13.0)	20.29	1.40 (1.04, 1.89)	1.29 (0.95, 1.74)
<7	5 to ≤30	45 (9.4)	15.21	1.03 (0.75, 1.43)	0.90 (0.65, 1.25)
7 to <9	5 to ≤30	34 (8.6)	14.36	0.94 (0.66, 1.36)	1.03 (0.72, 1.49)
≥9	5 to ≤30	3 (6.1)	11.58	0.63 (0.20, 1.96)	0.53 (0.17, 1.66)
<7	>30	191 (12.4)	20.26	1.38 (1.13, 1.68)	1.19 (0.97, 1.46)
7 to <9	>30	166 (9.7)	15.19	1.06 (0.86, 1.30)	1.06 (0.87, 1.31)
≥9	>30	28 (9.2)	15.77	1.01 (0.68, 1.49)	0.94 (0.63, 1.40)

a Adjusted for gender, age (categorical), marital status, education level, area of residence, smoking status, drinking status, BMI, social activities, baseline chronic condition status and sleep quality.

Of the original 9,753 participants, 1,073 (11.0%) developed HL. In the unadjusted model, participants with short duration of sleep (<5 h/night) were significantly associated with a higher incidence of HL compared with the 7 to <9 h/night group (HR: 1.99, 95% CI: 1.68, 2.36). The risk was attenuated yet the positive association remains with full adjustment (HR: 1.45, 95% CI: 1.20, 1.75). A similar association was discovered in the analysis of total sleep duration (Supplementary Table S4). In terms of midday napping, moderate nappers (5 to ≤30 min) had lower risks of HL compared with non-nappers after adjusting for confounders including sleep quality (HR: 0.80, 95% CI: 0.63, 1.00). Long nocturnal sleep (≥ 9 h/night) and long napping (> 30 min) were not significantly associated with HL.

**Figure 2:**
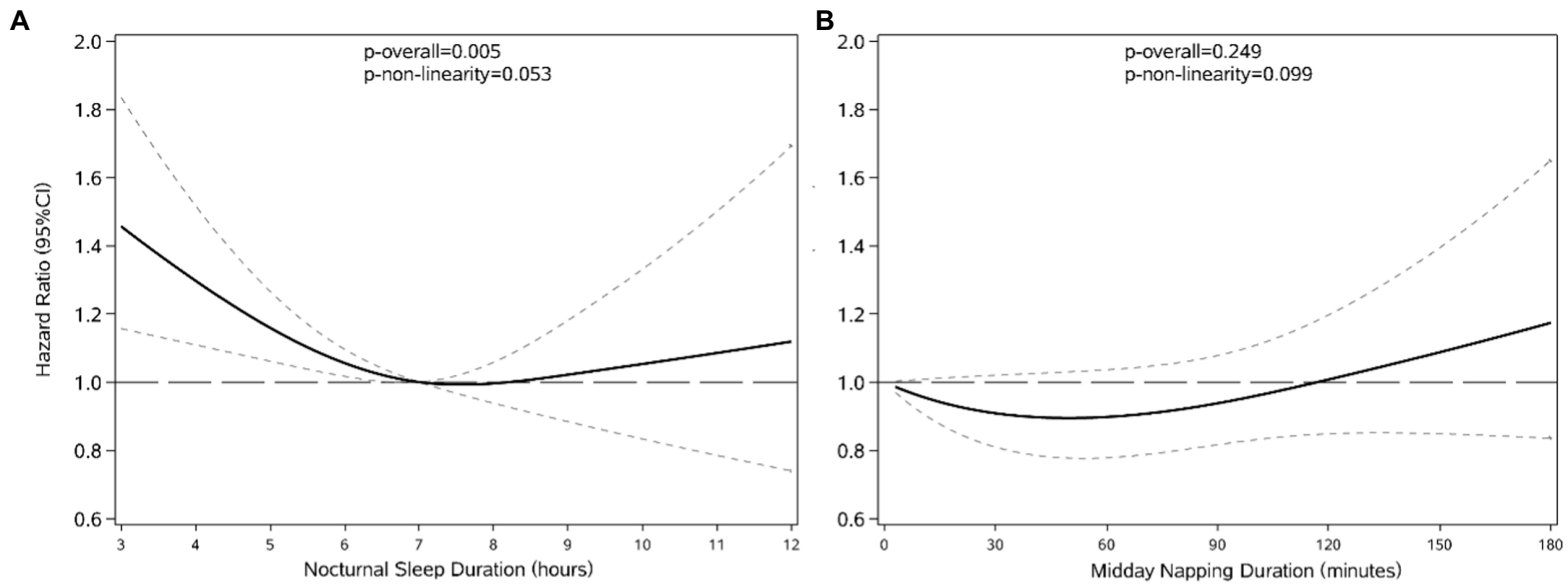
The shape of the association between nocturnal sleep duration **(A)**, midday napping **(B)** and the development of HL. Lines with short dashes represent the pointwise 95% confidence intervals for the fitted nonlinear trend (solid line). The horizontal line with long dashes represents the reference line (hazard ratio: 1). Data were adjusted for all variables. The reference group was 7 h/night for nocturnal sleep duration **(A)** and 0 min for midday napping duration **(B)**.

Restricted cubic splines was used to assess and visualize the relationship between sleep duration and HL. Figure 2 shows a reverse J-shaped dose–response association between nocturnal sleep and the hazards of HL after adjusting all covariates (*P* of nonlinear = 0.053). Compared with participants who slept 7 h/night, the hazards of HL increased with shorter or longer sleep at night. However, we did not observe a nonlinear association of midday napping (*P* of nonlinear = 0.099) with HL. Compared with non-nappers, the HR initially decreased and peaked at napping of about 45 min, then slowly increased at longer naps, while HRs remained non-significant for all napping durations.

For the analysis of joint association, relative to non-nappers with moderate nocturnal sleep duration, individuals who had sleep duration < 5 h/night and napping ≤ 5 min extended a higher hazard of HL (HR: 1.27, 95% CI: 1.06, 1.52). Short midday nappers with short or long duration of nocturnal sleep had a lower hazard of HL (HR: 0.90, 95% CI: 0.65, 1.25 for < 7 h/night; HR: 0.53, 95% CI: 0.17, 1.66 for ≥ 9 h/night), although not statistically significant.

### 3.3. Association between changes in sleep characteristics and hearing loss

The effects of changes in duration of nocturnal sleep and midday napping are presented in Table 3, with the lowest incidence of HL in the population with moderate time of sleep at night (8.1%) and napping (7.9%). Compared with the reference group, the risks of HL augmented by 29% among persons who consistently reported short nocturnal sleep (HR: 1.29, 95% CI: 1.07, 1.55), by 25% among persons who reported sleep duration increased from short to moderate (HR: 1.25, 95% CI: 1.00, 1.56). Additionally, persons whose nocturnal sleep duration increased from short to long reported the highest HL risk (HR: 1.73, 95% CI: 1.21, 2.48).

**Table 3:** Development of hearing loss by changes in subcategories of nocturnal sleep duration and midday napping duration.

Baseline	Follow-up	Case (%)	Crude incidence (1,000 person-year)	Adjusted HR^a^ (95%CI)
*Nocturnal sleep duration (hours)*				
<7	<7	397 (12.9)	20.44	1.29 (1.07, 1.55)
<7	7 to <9	150 (12.1)	19.21	1.25 (1.00, 1.56)
<7	≥9	37 (18.9)	31.12	1.73 (1.21, 2.48)
7 to <9	<7	178 (10.6)	16.49	1.20 (0.97, 1.48)
7 to <9	7 to <9	179 (8.1)	12.70	1 (ref)
7 to <9	≥9	47 (12.0)	19.43	1.21 (0.88, 1.67)
≥9	<7	20 (9.6)	15.50	0.93 (0.59, 1.48)
≥9	7 to <9	43 (10.8)	16.90	1.25 (0.90, 1.75)
≥9	≥9	22 (13.6)	22.27	1.37 (0.88, 2.14)
*Midday napping duration (minutes)*				
≤5	≤5	394 (12.3)	19.35	1 (ref)
≤5	5 to ≤30	55 (13.3)	22.00	1.16 (0.87, 1.54)
≤5	>30	157 (10.6)	16.50	0.88 (0.73, 1.06)
5 to ≤30	≤5	28 (10.3)	16.31	0.89 (0.60, 1.30)
5 to ≤30	5 to ≤30	25 (7.9)	13.12	0.76 (0.51, 1.14)
5 to ≤30	>30	29 (8.7)	14.82	0.71 (0.49, 1.04)
>30	≤5	109 (11.3)	18.93	0.96 (0.78, 1.19)
>30	5 to ≤30	25 (8.0)	13.48	0.73 (0.48, 1.09)
>30	>30	251 (11.0)	17.32	0.97 (0.82, 1.14)

a Adjusted for gender, age (categorical), marital status, education level, area of residence, smoking status, drinking status, BMI, social activities, baseline chronic condition status and sleep quality.

The risks of incident HL were neither considerably elevated nor reduced by changes in duration of midday napping. Modestly lower risks were observed in participants who napped for the consistently moderate duration (HR: 0.76, 95% CI: 0.51, 1.14), increased duration from 5 to ≤30 min (HR: 0.71, 95% CI: 0.49, 1.04) or decreased duration from > 30 min (HR: 0.73, 95% CI: 0.48, 1.09).

### 3.4. Cox generalized additive models and bivariate surface diagrams

**Figure 3:**
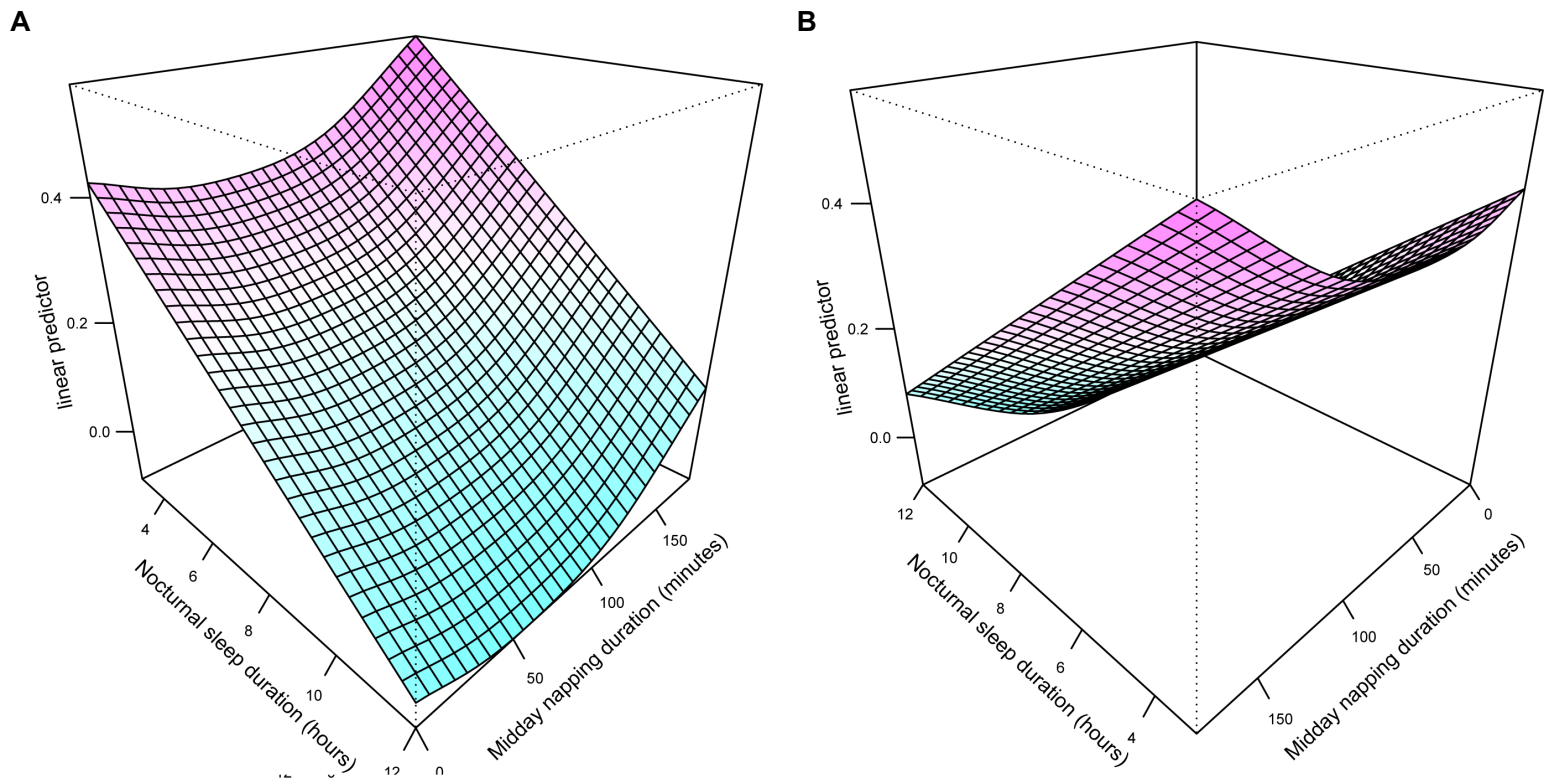
Different angles of exposure-response surface plots of the combined effects of nocturnal sleep duration **(A)** and midday napping duration **(B)**.

A U-shaped association between napping time and HL risk existed for the same nocturnal sleep time was shown in bivariate surface diagrams. With the extended napping duration, the risk of HL events declined until around 45 min and then rose rapidly, especially when napping duration ranged from 100 to 180 min. Longer nocturnal sleep enhanced HL risk regardless of napping. Overall, the bivariate response diagrams suggest a significant synergistic effect between nocturnal sleep and midday napping (Figure 3).

### 3.5. Stratified analysis and sensitivity analysis

The results show that among non-nappers, those who slept < 7 h/night had a significantly higher hazard of HL (HR: 1.26, 95% CI: 1.05, 1.51), while longer or shorter nocturnal sleep duration did not improve the HL risk across other categories of napping (Table 4). In addition, the moderate midday napping may reduce the risk of HL incidence for people who slept too short (HR: 0.72, 95% CI: 0.53, 0.99) or too long (HR: 0.42, 95% CI: 0.13, 1.36) at night (Table 5).

**Table 4:** Association between nocturnal sleep duration and hearing loss stratified by midday napping duration.

Midday napping duration (minutes)	Nocturnal sleep duration (hours)	Case (%)	Crude incidence (1,000 person-year)	Adjusted HR^a^ (95%CI)
≤5	<7	348 (14.0)	22.11	1.26 (1.05, 1.51)
	7 to <9	204 (9.3)	14.69	1 (ref)
	≥9	54 (13.0)	20.29	1.27 (0.94, 1.72)
5 to ≤30	<7	45 (9.4)	15.21	0.99 (0.61, 1.63)
	7 to <9	34 (8.6)	14.36	1 (ref)
	≥9	3 (6.1)	11.58	0.58 (0.17, 1.93)
>30	<7	191 (12.4)	20.26	1.21 (0.90, 1.40)
	7 to <9	166 (9.7)	15.19	1 (ref)
	≥9	28 (9.2)	15.77	0.85 (0.57, 1.28)

a Adjusted for gender, age (categorical), marital status, education level, area of residence, smoking status, drinking status, BMI, social activities, baseline chronic condition status and sleep quality.

**Table 5:** Association between midday napping duration and hearing loss stratified by nocturnal sleep duration.

Nocturnal sleep duration (hours)	Midday napping duration (minutes)	Case (%)	Crude incidence (1,000 person-year)	Adjusted HR^a^ (95%CI)
<7	≤5	348 (14.0)	22.11	1 (ref)
	5 to ≤30	45 (9.4)	15.21	0.72 (0.53, 0.99)
	>30	191 (12.4)	20.26	0.93 (0.78, 1.11)
7 to <9	≤5	204 (9.3)	14.69	1 (ref)
	5 to ≤30	34 (8.6)	14.36	1.00 (0.69, 1.44)
	>30	166 (9.7)	15.19	1.07 (0.98, 1.52)
≥9	≤5	54 (13.0)	20.29	1 (ref)
	5 to ≤30	3 (6.1)	11.58	0.42 (0.13, 1.36)
	>30	28 (9.2)	15.77	0.81 (0.50, 1.30)

a Adjusted for gender, age (categorical), marital status, education level, area of residence, smoking status, drinking status, BMI, social activities, baseline chronic condition status and sleep quality.

Sensitivity analyses were performed by excluding people with BMI ≥ 30 kg/m^2^, reclassifying time of nocturnal sleep into three groups and resetting 7–8 h/night as the reference group. Both dropping the obese individuals and resetting 7–8 h/night as the reference group did not change the direction and magnitude of association (Supplementary Table S6, S8). Combination of < 5, 5 to <6 and 6 to <7 groups may weaken the strength of the association (Supplementary Table S7). Additionally, the results from the multiply imputed datasets were similar to estimates obtained from complete case analysis (Supplementary Table S9).

## 4. Discussion

We discovered a significant association of short nocturnal sleep duration (<5 h/night) with subjective poor HL. Furthermore, moderate midday napping duration (5 to ≤30 min) may decrease risk of incident HL. The associations were independent of gender, age, marital status, education level, area of residence, smoking status, drinking status, BMI, social participation, baseline health condition and sleep quality.

A few population-based studies provided insights into the heterogeneous effects of nocturnal sleep duration (26–29). Some studies reported that long duration of nocturnal sleep could be a sign of high HL risk (26, 27). A recent article revealed an association between nocturnal sleep with ≤ 4 h (OR: 1.53, 95%CI: 1.31, 1.80) and HL in comparison to the 7 h group (26). Wu et al. (27) found that among the Chinese participants, sleep duration < 6 h/night was positively associated with high-frequency HL (OR: 1.46, 95%CI: 1.05, 2.03) and speech-frequency HL (OR: 1.38, 95%CI: 1.04, 1.83). The results of our research supported that people who slept with shorter time may have a higher risk of HL incidences by longitudinal analysis. Analysis of total sleep duration shows similar impacts. The pathophysiological mechanisms leading to the association are not fully clear. Inadequate sleep duration might affect fluid absorption and ion homeostasis and destruct hair cells in the cochlea by inflammatory cytokines and thus impair sensory transduction (12). Change of hippocampal structure is another potential procedure, by which sleep disturbed neural input from the central auditory system (11, 13). Also, sleep curtailment strikingly altered metabolic and endocrine function, causing eustachian tube dysfunction and poorer nerve conduction (45, 46). However, several other studies confirmed the association between short time of nocturnal sleep and HL (28, 29). The evidence from National Health and Nutrition Examination Survey (NHANES) demonstrated that every additional hour of sleep duration exceeded 8 h was marginally associated with a 2.89 dB higher pure-tone average (95% CI: 0.02, 5.76) on high-frequency hearing level (28). A Japanese study observed that compared with 6 h, prolonged sleep duration was significantly associated with HL (29). In discordance with findings in cross-sectional analysis, we merely observed the insignificantly increased risk of HL with longer nocturnal sleep lasted in the RCS plot. The disparities could be plausible given those different definitions of nocturnal sleep and the reference group, as well as different sources and sample sizes. Another possible reason is bias caused by the bidirectional relationship between sleep duration and HL, according to a population-based study in Israel, which suggested that subjects with pre-existing hearing impairment showed greater difficulty in maintaining sleep (47).

We found a modest beneficial effect of moderate midday napping (5 to ≤30 min) on incident HL after adjustment for sleep quantity and nocturnal sleep, implying its independent function. Epidemiological researches concerning the HL events in midday napping are still insufficient. However, several experimental studies accounted for its positive influence on unhealthy adverse events. Daytime sleep progressively increased parasympathetic activity as non-rapid eye movement sleep deepened, which could help to recover from hearing-related stress (48, 49). Besides, napping contributed to stress-releasing and immune responses as a countermeasure to the detrimental health consequences of sleep debt (50).

Notably, despite no significant interactions, we observed a significant joint effect that sleeping < 7 h/night in conjunction with ≤ 5 min of midday napping rose the risk of HL. Our results of stratified analysis supported that moderate midday napping exhibited a protective effect on those who slept inadequately at night. Bivariate surface diagrams show higher hazards of HL in pretty short nocturnal sleep without napping. This may be due to extremely short duration of sleep and no midday napping affecting hearing shared the common pathway, including worse subjective alertness and cognition and greater fatigue (18, 51). Another reason is that the above-mentioned sleep pattern reflects reduced total sleep duration.

Changes in duration of nocturnal sleep and napping have been linked to a variety of medical condition (23–25). In our sample, the change from baseline short sleep at night to other groups remarkably raised the risk of HL incidence. Relative to persistent short nocturnal sleep, sleep duration that shifted from baseline short to long, meaning disruption of sleep homeostatic processes, had a stronger effect on incident HL. One possible mechanism is the mutual relationship between sleep homeostat and the circadian phase (52). Also, activation of pro-inflammatory pathways may present a mechanism by which change in nocturnal sleep affect hearing (53). Although lack of statistical significance, midday napping change towards 5 to ≤30 min produced protective effects.

The main strengths of this study were demonstrated as follows. First, previous researches were cross-sectional and therefore causality and direction of effects cannot be elucidated. In addition, they majorly focused on a relatively small sample of general adults or the older-aged population. In contrast, we included a large sample containing middle-and old-aged adults from a nationally representative database, emphasizing not only separate effects of baseline sleep characteristics but also influences of their longitudinal changes on sequent HL. Second, the combined use of multivariable regressions and CGAMs could better illustrate the joint effects in a flexible and intuitionistic manner. Third, the application of RCS avoided insufficient statistical power caused by the categorization of sleep duration and disclosed the underlying non-linear correlation.

There are several limitations to our study. Health condition, sleep characteristics and hearing status were collected by self-report, which may lead to subjective bias, recall bias and loss of detailed data including sleep latency and sleep efficiency (54). Though previous studies have validated the accuracy of self-reported health condition in CHARLS (55, 56). Self-reported sleep duration agreed with objective measurement is considered as a simple and fast method (26). A cross-sectional study compared the subjective poor hearing to objective moderate to severe HL and observed similar prevalence (57). Although the accuracy of this definition still needs further verification, hearing measurement by a single-item question makes participants feel easy to answer and had been recommended for application in observational studies (58–60). It should be noted that self-reported HL, which represents the incidence of disturbance of hearing function, tends to underestimate or overestimate compared to objective measurements due to age, sex, physical and mental health conditions, etc. (61–63). The observed incidence of HL in our study was lower than total prevalence reported by another study in China (1). However, results of this study were closer to prevalence of disabling HL ranged from 8.0 to 16.1% in several surveys (64–67). Therefore, we applied the definition in the present study to minimize potential measurement error and focus on poor HL. Five-way stratification of sleep duration may introduce exposure classification bias. Therefore, in addition to considering extreme sleep duration and re-categorized it into three groups, we also employed RCS to lessen the possibility of bias. Results show consistency. It is widely confirmed in the existing literature that ambient noise, OSAH and snoring are independent risk factors of HL, which the CHARLS dataset did not record (41, 42, 68–70). However, even after controlling for noise exposure, the association between short sleep duration and HL remained significant (71). Analysis with the exclusion of obese persons after controlling for sleep quality did not alter the results primarily indicating the limited bias of OSAH and snoring. Besides, effects on health may differ between types of midday napping, which our study did not consider (72). More studies on large representative samples are needed to better understand the impacts of nocturnal sleep, midday napping and sleep patterns on hearing status in the future.

## 5. Conclusion

Our study provided community-based and longitudinal evidence on the impact of short nocturnal sleep duration, moderate napping and their changes on HL in middle-aged and older Chinese adults. The results highlighted the harmful effect of sleep patterns of short nocturnal sleep without napping. Monitoring and intervening in unhealthy sleep characteristics could be a potentially effective strategy for the management of HL.

## Data availability statement

Publicly available datasets were analyzed in this study. This data can be found at: https://charls.charlsdata.com/pages/data/111/zh-cn.html.

## Ethics statement

The studies involving human participants were reviewed and approved by Biomedical Ethics Review Committee of Peking University. The patients/participants provided their written informed consent to participate in this study.

## Author contributions

XC, ZL, XG, ND, CH, and YL contributed to conception, design, methodology and investigation of the study. ZL and JC organized the database. XC, ZL, and RL performed the statistical analysis. XC and ZL wrote the first draft of the manuscript. YY, GQ, and JC participated in resources, manuscript revision and funding acquisition. All authors contributed to the article and approved the submitted version.

## Funding

This study was funded by the National Natural Science Foundation of China (grants 82173612 and 82273730); Shanghai Rising-Star Program (21QA1401300); Shanghai Municipal Natural Science Foundation (22ZR1414900); Shanghai Municipal Science and Technology Major Project (ZD2021CY001).

## Conflict of interest

The authors declare that the research was conducted in the absence of any commercial or financial relationships that could be construed as a potential conflict of interest.

## Publisher’s note

All claims expressed in this article are solely those of the authors and do not necessarily represent those of their affiliated organizations, or those of the publisher, the editors and the reviewers. Any product that may be evaluated in this article, or claim that may be made by its manufacturer, is not guaranteed or endorsed by the publisher.

## Abbreviations

BMI: body mass index
CHARLS: China health and retirement longitudinal study
CI: confidence interval
CGAM: Cox generalized additive model
DAG: directed acyclic graph
DALYs: disability-adjusted life-years
dB: decibel
HR: hazards ratio
HL: hearing loss
IQR: interquartile range
NHANES: National Health and Nutrition Examination Survey
OSAH: obstructive sleep apnea-hypopnea
OR: odds ratio
RCS: restricted cubic splines

## References

[1] Gong R Hu X Gong C Long M Han R Zhou L. Hearing loss prevalence and risk factors among older adults in China. Int J Audiol. (2018) 57:354–9. doi: 10.1080/14992027.2017.1423404 29400111

[2] Collaborators GDaI. Global burden of 369 diseases and injuries in 204 countries and territories, 1990-2019: a systematic analysis for the global burden of disease study 2019. Lancet. (2020) 396:1204–22. doi: 10.1016/S0140-6736(20)30925-9, PMID: 33069326 PMC7567026

[3] Amieva H Ouvrard C Giulioli C Meillon C Rullier L Dartigues JF. Self-reported hearing loss, hearing aids, and cognitive decline in elderly adults: a 25-year study. J Am Geriatr Soc. (2015) 63:2099–104. doi: 10.1111/jgs.13649, PMID: 26480972

[4] McKee MM Stransky ML Reichard A. Hearing loss and associated medical conditions among individuals 65 years and older. Disabil Health J. (2018) 11:122–5. doi: 10.1016/j.dhjo.2017.05.007, PMID: 28596096

[5] Deal JA Betz J Yaffe K Harris T Purchase-Helzner E Satterfield S. Hearing impairment and incident dementia and cognitive decline in older adults: the health ABC study. J Gerontol A Biol Sci Med Sci. (2017) 72:glw069–9. doi: 10.1093/gerona/glw069, PMID: 27071780 PMC5964742

[6] Shukla A Harper M Pedersen E Goman A Suen JJ Price C. Hearing loss, loneliness, and social isolation: a systematic review. Otolaryngol Head Neck Surg. (2020) 162:622–33. doi: 10.1177/0194599820910377, PMID: 32151193 PMC8292986

[7] Wang J Liu D Tian E Guo ZQ Chen JY Kong WJ. Hearing impairment with cognitive decline increases all-cause mortality risk in Chinese adults aged 65 years or older: a population-based longitudinal study. Front Aging Neurosci. (2022) 14:865821. doi: 10.3389/fnagi.2022.865821, PMID: 35813959 PMC9263259

[8] Ning H Zhang H Xie Z Jiang W Xie S. Relationship of hearing impairment, social participation and depressive symptoms to the incidence of frailty in a community cohort. J Am Geriatr Soc. (2022):1–10. doi: 10.1111/jgs.18164, PMID: 36504135

[9] Walling AD Dickson GM. Hearing loss in older adults. Am Fam Physician. (2012) 173:293–9. doi: 10.1001/jamainternmed.2013.1868, PMID: 22962895

[10] Bah TM Goodman J Iliff JJ. Sleep as a therapeutic target in the aging brain. Neurotherapeutics. (2019) 16:554–68. doi: 10.1007/s13311-019-00769-6, PMID: 31376067 PMC6694322

[11] Noh HJ Joo EY Kim ST Yoon SM Koo DL Kim D. The relationship between hippocampal volume and cognition in patients with chronic primary insomnia. J Clin Neurol. (2012) 8:130–8. doi: 10.3988/jcn.2012.8.2.130, PMID: 22787497 PMC3391618

[12] Jung JH Kim M Lee SJ Lee E Lee SA Lee JD. Effect of sleep deprivation on hearing levels in rats. Int J Pediatr Otorhinolaryngol. (2018) 112:169–75. doi: 10.1016/B978-0-444-53702-7.00007-5, PMID: 30055728

[13] Ma W Zhang Y Li X Liu S Gao Y Yang J. High-frequency hearing loss is associated with anxiety and brain structural plasticity in older adults. Front Aging Neurosci. (2022) 14:821537. doi: 10.3389/fnagi.2022.821537, PMID: 35360202 PMC8961435

[14] Leng Y Cappuccio FP Surtees PG Luben R Brayne C Khaw KT. Daytime napping, sleep duration and increased 8-year risk of type 2 diabetes in a British population. Nutr Metab Cardiovasc Dis. (2016) 26:996–1003. doi: 10.1016/j.numecd.2016.06.006, PMID: 27484757 PMC5084679

[15] Chen Y Kartsonaki C Clarke R Guo Y Yu C Bian Z. Characteristics and correlates of sleep duration, daytime napping, snoring and insomnia symptoms among 0.5 million Chinese men and women. Sleep Med. (2018) 44:67–75. doi: 10.1016/j.sleep.2017.11.1131IF, PMID: 29530372 PMC5869948

[16] Xiao Q Hale L. Neighborhood socioeconomic status, sleep duration, and napping in middle-to-old aged US men and women. Sleep. (2018) 41:41. doi: 10.1093/sleep/zsy076, PMID: 29697844 PMC6047422

[17] Wang C Bangdiwala SI Rangarajan S Lear SA AlHabib KF Mohan V. Association of estimated sleep duration and naps with mortality and cardiovascular events: a study of 116 632 people from 21 countries. Eur Heart J. (2019) 40:1620–9. doi: 10.1093/eurheartj/ehy695, PMID: 30517670 PMC6528160

[18] Milner CE Cote KA. Benefits of napping in healthy adults: impact of nap length, time of day, age, and experience with napping. J Sleep Res. (2009) 18:272–81. doi: 10.1111/j.1365-2869.2008.00718.x, PMID: 19645971

[19] Foley DJ Vitiello MV Bliwise DL Ancoli-Israel S Monjan AA Walsh JK. Frequent napping is associated with excessive daytime sleepiness, depression, pain, and Nocturia in older adults: findings from the National Sleep Foundation ‘2003 sleep in America’ poll. Am J Geriatr Psychiatry. (2007) 15:344–50. doi: 10.1097/01.JGP.0000249385.50101.67, PMID: 17384317

[20] Devine JK Wolf JM. Integrating nap and night-time sleep into sleep patterns reveals differential links to health-relevant outcomes. J Sleep Res. (2016) 25:225–33. doi: 10.1111/jsr.12369, PMID: 26718988

[21] Bai Y Li X Wang K Chen S Wang S Chen Z. Association of shift-work, daytime napping, and nighttime sleep with cancer incidence and cancer-caused mortality in Dongfeng-tongji cohort study. Ann Med. (2016) 48:641–51. doi: 10.1080/07853890.2016.1217037, PMID: 27558895

[22] Li X Pang X Liu Z Zhang Q Sun C Yang J. Joint effect of less than 1 h of daytime napping and seven to 8 h of night sleep on the risk of stroke. Sleep Med. (2018) 52:180–7. doi: 10.1016/j.sleep.2018.05.011, PMID: 30408698

[23] Cheng GH Malhotra R Østbye T Chan A Ma S Lo JC. Changes in nocturnal sleep and daytime nap durations predict all-cause mortality among older adults: the panel on health and ageing of Singaporean elderly. Sleep. (2018) 41:41. doi: 10.1093/sleep/zsy087, PMID: 29722881

[24] Hua J Sun H Shen Y. Improvement in sleep duration was associated with higher cognitive function: a new association. Aging (Albany NY). (2020) 12:20623–44. doi: 10.18632/aging.103948, PMID: 33082298 PMC7655193

[25] Zhou L Yu K Yang L Wang H Xiao Y Qiu G. Sleep duration, midday napping, and sleep quality and incident stroke: the Dongfeng-Tongji cohort. Neurology. (2020) 94:e345–56. doi: 10.1212/WNL.0000000000008739, PMID: 31827003

[26] Rong H Wang X Lai X Yu W Fei Y. Associations between sleep duration and sensory impairments among older adults in China. Front Aging Neurosci. (2022) 14:910231. doi: 10.3389/fnagi.2022.910231, PMID: 35754970 PMC9228799

[27] Wu E Ni J Zhu Z Xu H Ci J Tao L. Association of sleep duration and noise exposure with hearing loss among Chinese and American adults: two cross-sectional studies. BMJ Open. (2022) 12:e062535. doi: 10.1136/bmjopen-2022-062535, PMID: 36127089 PMC9490609

[28] Jiang K Spira AP Reed NS Lin FR Deal JA. Sleep characteristics and hearing loss in older adults: the National Health and nutrition examination survey 2005-2006. J Gerontol A Biol Sci Med Sci. (2022) 77:632–9. doi: 10.1093/gerona/glab214, PMID: 34302481 PMC9122752

[29] Nakajima K Kanda E Hosobuchi A Suwa K. Subclinical hearing loss, longer sleep duration, and cardiometabolic risk factors in Japanese general population. Int J Otolaryngol. (2014) 2014:218218. doi: 10.1155/2014/218218, PMID: 25214844 PMC4158149

[30] Zhao Y Hu Y Smith JP Strauss J Yang G. Cohort profile: the China health and retirement longitudinal study (CHARLS). Int J Epidemiol. (2014) 43:61–8. doi: 10.1093/ije/dys203, PMID: 23243115 PMC3937970

[31] Hirshkowitz M Whiton K Albert SM Alessi C Bruni O Don Carlos L. National Sleep Foundation’s sleep time duration recommendations: methodology and results summary. Sleep Health. (2015) 1:40–3. doi: 10.1016/j.sleh.2014.12.010, PMID: 29073412

[32] Jung KI Song CH Ancoli-Israel S Barrett-Connor E. Gender differences in nighttime sleep and daytime napping as predictors of mortality in older adults: the rancho Bernardo study. Sleep Med. (2013) 14:12–9. doi: 10.1016/j.sleep.2012.06.004, PMID: 22951185 PMC3542414

[33] Igarashi A Aida J Yamamoto T Hiratsuka Y Kondo K Osaka K. Associations between vision, hearing and tooth loss and social interactions: the JAGES cross-sectional study. J Epidemiol Community Health. (2021) 75:jech-2020-214545–6. doi: 10.1136/jech-2020-214545, PMID: 32972921

[34] Guan L Liu Q Chen D Chen C Wang Z. Hearing loss, depression, and medical service utilization among older adults: evidence from China. Public Health. (2022) 205:122–9. doi: 10.1016/j.puhe.2022.01.025, PMID: 35278783

[35] Ma X Wei J Congdon N Li Y Shi L Zhang D. Longitudinal association between self-reported sensory impairments and episodic memory among older adults in China: a prospective cohort study. J Geriatr Psychiatry Neurol. (2022) 35:382–91. doi: 10.1177/08919887211006467, PMID: 33792435

[36] Wang Z Chen D Pan T Chen C Guan L. Hearing loss, depression and social participation of older adults: evidence from the China health and retirement longitudinal study. Geriatr Gerontol Int. (2022) 22:529–35. doi: 10.1111/ggi.14413, PMID: 35674053

[37] Kuo PL Huang AR Ehrlich JR Kasper J Lin FR McKee MM. Prevalence of concurrent functional vision and hearing impairment and association with dementia in community-dwelling Medicare beneficiaries. JAMA Netw Open. (2021) 4:e211558. doi: 10.1001/jamanetworkopen.2021.1558, PMID: 33739429 PMC8601132

[38] Wang R Chen Z Zhou Y Shen L Zhang Z Wu X. Melancholy or mahjong? Diversity, frequency, type, and rural-urban divide of social participation and depression in middle-and old-aged Chinese: a fixed-effects analysis. Soc Sci Med. (2019) 238:112518. doi: 10.1016/j.socscimed.2019.112518 31473574

[39] Li M Wang N Dupre ME. Association between the self-reported duration and quality of sleep and cognitive function among middle-aged and older adults in China. J Affect Disord. (2022) 304:20–7. doi: 10.1016/j.jad.2022.02.039, PMID: 35176346

[40] Pedersen EJ Miller DL Simpson GL Ross N. Hierarchical generalized additive models in ecology: an introduction with mgcv. Peer J. (2019) 7:e6876. doi: 10.7717/peerj.6876, PMID: 31179172 PMC6542350

[41] Chen CK Shen SC Lee LA Sun MH Chen NH Chuang LP. Idiopathic sudden sensorineural hearing loss in patients with obstructive sleep apnea. Nat Sci Sleep. (2021) 13:1877–85. doi: 10.2147/NSS.S331880, PMID: 34703345 PMC8526947

[42] Lu CT Lee LA Lee GS Li HY. Obstructive sleep apnea and auditory dysfunction-does snoring sound play a role? Diagnostics (Basel). (2022) 12:2734. doi: 10.3390/diagnostics12102374 36292063 PMC9600079

[43] McNicholas WT. Diagnosis of obstructive sleep apnea in adults. Proc Am Thorac Soc. (2008) 5:154–60. doi: 10.1513/pats.200708-118MG 18250207

[44] Hamilton GS Joosten SA. Obstructive sleep apnoea and obesity. Aust Fam Physician. (2017) 46:460–3. 28697288

[45] Spiegel K Leproult R Van Cauter E. Impact of sleep debt on metabolic and endocrine function. Lancet. (1999) 354:1435–9. doi: 10.1016/S0140-6736(99)01376-8, PMID: 10543671

[46] Cherian KE Kapoor N Mathews SS Paul TV. Endocrine glands and hearing: auditory manifestations of various endocrine and metabolic conditions. Indian J Endocrinol Metab. (2017) 21:464–9. doi: 10.4103/ijem.IJEM_10_17, PMID: 28553606 PMC5434734

[47] Test T Canfi A Eyal A Shoam-Vardi I Sheiner EK. The influence of hearing impairment on sleep quality among workers exposed to harmful noise. Sleep. (2011) 34:25–30. doi: 10.1093/sleep/34.1.25, PMID: 21203368 PMC3001791

[48] Wang Y Zekveld AA Naylor G Ohlenforst B Jansma EP Lorens A. Parasympathetic nervous system dysfunction, as identified by pupil light reflex, and its possible connection to hearing impairment. PLoS One. (2016) 11:e0153566. doi: 10.1371/journal.pone.0153566, PMID: 27089436 PMC4835104

[49] AlQatari AA Alturki JA Abdulali KA Alhumud DA Alibrahim MA Alarab YA. Changes in heart rate variability and Baroreflex sensitivity during daytime naps. Nat Sci Sleep. (2020) 12:661–9. doi: 10.2147/NSS.S270191, PMID: 33061723 PMC7520661

[50] Faraut B Nakib S Drogou C Elbaz M Sauvet F De Bandt JP. Napping reverses the salivary interleukin-6 and urinary norepinephrine changes induced by sleep restriction. J Clin Endocrinol Metab. (2015) 100:E416–26. doi: 10.1210/jc.2014-2566, PMID: 25668196

[51] Tietzel AJ Lack LC. The short-term benefits of brief and long naps following nocturnal sleep restriction. Sleep. (2001) 24:293–300. doi: 10.1093/sleep/24.3.293, PMID: 11322712

[52] Borbély AA Daan S Wirz-Justice A Deboer T. The two-process model of sleep regulation: a reappraisal. J Sleep Res. (2016) 25:131–43. doi: 10.1111/jsr.12371, PMID: 26762182

[53] Patel SR Zhu X Storfer-Isser A Mehra R Jenny NS Tracy R. Sleep duration and biomarkers of inflammation. Sleep. (2009) 32:200–4. doi: 10.1093/sleep/32.2.200, PMID: 19238807 PMC2635584

[54] Van Den Berg JF Van Rooij FJ Vos H Tulen JH Hofman A Miedema HM. Disagreement between subjective and actigraphic measures of sleep duration in a population-based study of elderly persons. J Sleep Res. (2008) 17:295–302. doi: 10.1111/j.1365-2869.2008.00638.x, PMID: 18321246

[55] Yuan X Liu T Wu L Zou ZY Li C. Validity of self-reported diabetes among middle-aged and older Chinese adults: the China health and retirement longitudinal study. BMJ Open. (2015) 5:e006633. doi: 10.1136/bmjopen-2014-006633, PMID: 25872937 PMC4401856

[56] Ning M Zhang Q Yang M. Comparison of self-reported and biomedical data on hypertension and diabetes: findings from the China health and retirement longitudinal study (CHARLS). BMJ Open. (2016) 6:e009836. doi: 10.1136/bmjopen-2015-009836, PMID: 26729390 PMC4716227

[57] Tsimpida D Kontopantelis E Ashcroft D Panagioti M. Comparison of self-reported measures of hearing with an objective audiometric measure in adults in the English longitudinal study of ageing. JAMA Netw Open. (2020) 3:e2015009. doi: 10.1001/jamanetworkopen.2020.15009, PMID: 32852555 PMC7453309

[58] Nondahl DM Cruickshanks KJ Wiley TL Tweed TS Klein R Klein BEK. Accuracy of self-reported hearing loss. Audiology. (1998) 37:295–301. doi: 10.3109/00206099809072983 9776206

[59] Valete-Rosalino CM Rozenfeld S. Auditory screening in the elderly: comparison between self-report and audiometry. Braz J Otorhinolaryngol. (2005) 71:193–200. doi: 10.1016/s1808-8694(15)31310-0, PMID: 16446917 PMC9450545

[60] Ferrite S Santana VS Marshall SW. Validity of self-reported hearing loss in adults: performance of three single questions. Rev Saude Publica. (2011) 45:824–30. doi: 10.1590/s0034-89102011005000050, PMID: 21808834

[61] Tremblay KL Pinto A Fischer ME Klein BE Klein R Levy S. Self-reported hearing difficulties among adults with Normal audiograms: the beaver dam offspring study. Ear Hear. (2015) 36:e290–9. doi: 10.1097/AUD.0000000000000195, PMID: 26164105 PMC4824300

[62] Hämäläinen A Pichora-Fuller MK Wittich W Phillips NA Mick P. Self-report measures of hearing and vision in older adults participating in the Canadian longitudinal study of aging are explained by behavioral sensory measures, demographic, and social factors. Ear Hear. (2021) 42:814–31. doi: 10.1097/AUD.0000000000000992, PMID: 33741763

[63] Humes LE. Factors underlying individual differences in speech-recognition threshold (SRT) in noise among older adults. Front Aging Neurosci. (2021) 13:702739. doi: 10.3389/fnagi.2021.702739, PMID: 34290600 PMC8287901

[64] Liu YD Wang YR Xing WL Feng L Guo S Dai P. Prevalence and related factors of visual disability, hearing disability and comorbidity of visual and hearing disability among the elderly in China. Zhonghua Yi Xue Za Zhi. (2023) 103:436–41. doi: 10.3760/cma.j.cn112137-20221124-02485, PMID: 36775268

[65] Nicholas SO Koh EJ Wee SL Eikelboom RH Jayakody DMP Lin F. Peripheral hearing loss and its association with cognition among ethnic Chinese older adults. Dement Geriatr Cogn Disord. (2021) 50:394–400. doi: 10.1159/000519291, PMID: 34592737

[66] Bright T Shan X Xu J Liang J Xiao B Ensink R. Field-testing of a rapid survey method to assess the prevalence and causes of hearing loss in Gao'an, Jiangxi province. China Arch Public Health. (2020) 78:16. doi: 10.1186/s13690-020-0398-1, PMID: 32166026 PMC7059708

[67] Sindhusake D Mitchell P Smith W Golding M Newall P Hartley D. Validation of self-reported hearing loss. The Blue Mountains hearing study. Int J Epidemiol. (2001) 30:1371–8. doi: 10.1093/ije/30.6.1371, PMID: 11821349

[68] Hong O Kerr MJ Poling GL Dhar S. Understanding and preventing noise-induced hearing loss. Dis Mon. (2013) 59:110–8. doi: 10.1016/j.disamonth.2013.01.002 23507351

[69] Ekin S Turan M Arısoy A Gunbatar H Sunnetcioglu A Asker S. Is there a relationship between obstructive sleep apnea (OSA) and hearing loss? Med Sci Monit. (2016) 22:3124–8. doi: 10.12659/msm.897347, PMID: 27588548 PMC5019138

[70] Wang C Xu F Chen M Chen X Li C Sun X. Association of obstructive sleep Apnea–Hypopnea syndrome with hearing loss: a systematic review and meta-analysis. Front Neurol. (2022) 13:1017982. doi: 10.3389/fneur.2022.1017982, PMID: 36341085 PMC9626824

[71] Lim HM Kang W Park WJ Jang KH Ann JS Moon JD. Insomnia and hearing impairment among occupational noise exposed male workers. Ann Occup Environ Med. (2017) 29:36. doi: 10.1186/s40557-017-0195-7, PMID: 28824813 PMC5558741

[72] Mantua J Spencer RMC. Exploring the nap paradox: are mid-day sleep bouts a friend or foe? Sleep Med. (2017) 37:88–97. doi: 10.1016/j.sleep.2017.01.019, PMID: 28899546 PMC5598771

