# Supplementary material for: Association of nocturnal sleep duration and midday napping with subjective poor hearing among middle-aged and older adults in China
Source: Front Public Health. 2023 Apr 11;11:1160294. doi: 10.3389/fpubh.2023.1160294 (PMC10127254; doi:10.3389/fpubh.2023.1160294)
Supplement: Supplementary file 1 [file Data_Sheet_1.docx]

Supplementary Material

**
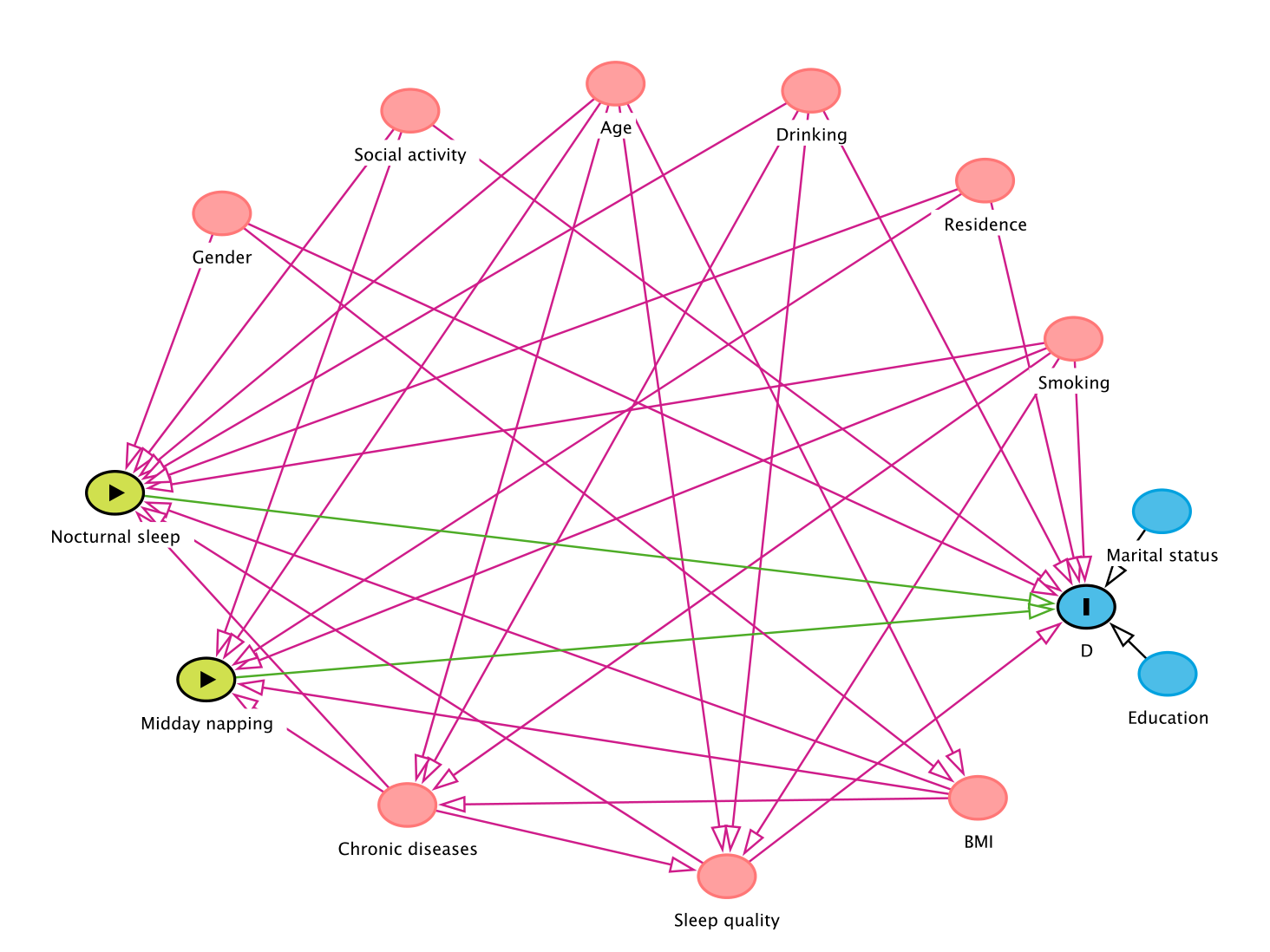
**

#### Supplementary Figure S1. Causal diagrams showing the relationship between variables

#### Supplementary Table S1. Characteristics of studied population at baseline by nocturnal sleep duration

| **Variables** | **Nocturnal sleep duration (hours)** | | | | | | | **P value** |
| --- | --- | --- | --- | --- | --- | --- | --- | --- |
|  | **<5** | **5-<6** | **6-<7** | | **7-<9** | | **≥9** |  |
|  | **N=1090** | **N=1230** | **N=2189** | | **N=4295** | | **N=769** |  |
| Male, n(%) | 456 (41.8) | 580 (47.2) | 1096 (50.1) | | 2076 (48.3) | | 350 (45.5) | <0.001 |
| Age, n(%) |  |  |  | |  | |  | <0.001 |
| <50 | 207 (19.0) | 268 (21.8) | 585 (26.7) | | 1328 (30.9) | | 223 (29.0) |  |
| <60 | 380 (34.9) | 472 (38.4) | 810 (37.0) | | 1557 (36.3) | | 264 (34.3) |  |
| <70 | 356 (32.7) | 374 (30.4) | 594 (27.1) | | 1054 (24.5) | | 191 (24.8) |  |
| **≥**70 | 147 (13.4) | 116 (9.4) | 200 (9.1) | | 356 (8.3) | | 91 (11.8) |  |
| BMI category, n(%) |  |  |  | |  | |  | <0.001 |
| <18.5 | 78 (7.2) | 72 (5.9) | 103 (4.7) | | 178 (4.1) | | 36 (4.7) |  |
| <24 | 611 (56.1) | 654 (53.2) | 1103 (50.4) | | 2155 (50.2) | | 423 (55.0) |  |
| <28 | 294 (27.0) | 371 (30.2) | 699 (31.9) | | 1385 (32.3) | | 219 (28.5) |  |
| **≥**28 | 107 (9.8) | 133 (10.8) | 284 (13.0) | | 577 (13.4) | | 91 (11.8) |  |
| Rural residents, n(%) | 699 (64.1) | 773 (62.9) | 1261 (57.6) | | 2651 (61.7) | | 532 (69.2) | <0.001 |
| Married, n(%) | 882 (80.9) | 1028 (83.6) | 1902 (86.9) | | 3740 (87.1) | | 644 (83.8) | <0.001 |
| Education level, n(%) |  |  | |  | |  | | <0.001 |
| Below primary school | 67 (6.1) | 123 (10.0) | 336 (15.4) | | 613 (14.3) | | 59 (7.7) |  |
| Primary school | 191 (17.5) | 269 (21.9) | 565 (25.8) | | 1079 (25.1) | | 151 (19.6) |  |
| Middle school | 259 (23.8) | 302 (24.6) | 523 (23.9) | | 1008 (23.5) | | 194 (25.2) |  |
| High school or above | 573 (52.6) | 536 (43.6) | 765 (35.0) | | 1595 (37.1) | | 365 (47.5) |  |
| Ever smoking, n(%) | 402 (36.9) | 496 (40.3) | 885 (40.4) | | 1656 (38.6) | | 289 (37.6) | 0.214 |
| Current drinking, n(%) | 347 (31.8) | 429 (34.9) | 822 (37.6) | | 1503 (35.0) | | 242 (31.5) | 0.004 |
| Types of social participation, n(%) |  |  |  | |  | |  | <0.001 |
| 0 | 545 (50.0) | 607 (49.4) | 1011 (46.2) | | 1894 (44.1) | | 365 (47.5) |  |
| 1 | 474 (43.5) | 516 (42.0) | 967 (44.2) | | 1978 (46.1) | | 345 (44.9) |  |
| 2 or more | 71 (6.5) | 107 (8.7) | 211 (9.6) | | 423 (9.9) | | 59 (7.7) |  |
| Hypertension, n(%) | 259 (23.8) | 296 (24.1) | 459 (21.0) | | 869 (20.2) | | 161 (20.9) | 0.013 |
| Diabetes, n(%) | 67 (6.1) | 80 (6.5) | 120 (5.5) | | 219 (5.1) | | 38 (4.9) | 0.281 |
| Stroke, n(%) | 25 (2.3) | 19 (1.5) | 41 (1.9) | | 55 (1.3) | | 4 (0.5) | 0.011 |
| Heart disease, n(%) | 141 (12.9) | 135 (11.0) | 204 (9.3) | | 374 (8.7) | | 50 (6.5) | <0.001 |
| Psychiatric disease, n(%) | 21 (1.9) | 18 (1.5) | 17 (0.8) | | 33 (0.8) | | 3 (0.4) | <0.001 |
| Memory-related disease, n(%) | 12 (1.1) | 13 (1.1) | 16 (0.7) | | 29 (0.7) | | 5 (0.7) | 0.478 |
| Dyslipidemia, n(%) | 97 (8.9) | 125 (10.2) | 196 (9.0) | | 371 (8.6) | | 59 (7.7) | 0.378 |
| Cancer, n(%) | 12 (1.1) | 14 (1.1) | 19 (0.9) | | 24 (0.6) | | 7 (0.9) | 0.168 |
| Chronic lung disease, n(%) | 130 (11.9) | 122 (9.9) | 168 (7.7) | | 315 (7.3) | | 51 (6.6) | <0.001 |
| Sleep quality, n(%) |  |  |  | |  | |  | <0.001 |
| Good | 404 (7.9) | 759 (14.8) | 3198 (62.5) | | 512 (10.0) | | 246 (4.8) |  |
| Fair | 686 (23.3) | 705 (24.0) | 1350 (45.9) | | 145 (4.9) | | 53 (1.8) |  |
| Bad | 757 (49.7) | 2297 (19.5) | 398 (26.2) | | 48 (3.2) | | 22 (1.5) |  |

Data are expressed as proportions for categorical variables. BMI, body mass index.

#### Supplementary Table S2. Characteristics of studied population at baseline by midday napping duration

| **Variables** | **Midday napping duration (minutes)** | | | **P value** |
| --- | --- | --- | --- | --- |
|  | **≤5** | **5-≤30** | **>30** |  |
|  | **N=5090** | **N=920** | **N=3563** |  |
| Male, n(%) | 2196 (43.1) | 393 (42.7) | 1969 (55.3) | <0.001 |
| Age, n(%) |  |  |  | 0.103 |
| <50 | 1384 (27.2) | 249 (27.1) | 978 (27.5) |  |
| <60 | 1909 (37.5) | 342 (37.2) | 1232 (34.6) |  |
| <70 | 1341 (26.4) | 240 (26.1) | 988 (27.7) |  |
| ≥70 | 1384 (27.2) | 249 (27.1) | 978 (27.5) |  |
| BMI category, n(%) |  |  |  | <0.001 |
| <18.5 | 279 (5.5) | 40 (4.4) | 148 (4.2) |  |
| <24 | 2749 (54.0) | 463 (50.3) | 1734 (48.7) |  |
| <28 | 1461 (28.7) | 298 (32.4) | 1209 (33.9) |  |
| ≥28 | 601 (11.8) | 119 (12.9) | 472 (13.3) |  |
| Rural residents, n(%) | 3260 (64.1) | 456 (59.6) | 2200 (61.8) | <0.001 |
| Married, n(%) | 4328 (85.0) | 801 (87.1) | 3067 (86.1) | 0.164 |
| Education level, n(%) |  |  |  | <0.001 |
| Below primary school | 535 (10.5) | 164 (17.8) | 499 (14.0) |  |
| Primary school | 1147 (22.5) | 234 (25.4) | 874 (24.5) |  |
| Middle school | 1232 (24.2) | 184 (20.0) | 870 (24.4) |  |
| High school or above | 2176 (42.8) | 338 (36.7) | 1320 (37.1) |  |
| Ever smoking, n(%) | 1846 (36.3) | 299 (32.5) | 1583 (44.4) | <0.001 |
| Current drinking, n(%) | 1586 (31.2) | 319 (34.7) | 1438 (40.4) | <0.001 |
| Types of social participation, n(%) |  |  |  | <0.001 |
| 0 | 2484 (48.8) | 386 (42.0) | 1552 (43.6) |  |
| 1 | 2211 (43.4) | 440 (47.8) | 1629 (45.7) |  |
| 2 or more | 395 (7.8) | 94 (10.2) | 382 (10.7) |  |
| Hypertension, n(%) | 973 (19.1) | 206 (22.4) | 865 (24.3) | <0.001 |
| Diabetes, n(%) | 225 (4.4) | 73 (7.9) | 226 (6.3) | <0.001 |
| Stroke, n(%) | 71 (1.4) | 14 (1.5) | 59 (1.7) | 0.617 |
| Heart disease, n(%) | 443 (8.7) | 109 (11.9) | 352 (9.9) | 0.006 |
| Psychiatric disease, n(%) | 48 (0.9) | 12 (1.3) | 32 (0.9) | 0.521 |
| Memory-related disease, n(%) | 36 (0.7) | 3 (0.3) | 36 (1.0) | 0.074 |
| Dyslipidemia, n(%) | 390 (7.7) | 104 (11.3) | 354 (9.9) | <0.001 |
| Cancer, n(%) | 43 (0.8) | 7(0.8) | 26 (0.7) | 0.833 |
| Chronic lung disease, n(%) | 404 (7.9) | 66 (7.2) | 316 (8.9) | 0.145 |
| Sleep quality, n(%) |  |  |  | <0.001 |
| Good | 2583 (50.8) | 458 (49.8) | 2074 (58.2) |  |
| Fair | 1619 (31.8) | 290 (31.5) | 1029 (28.9) |  |
| Bad | 888 (17.5) | 172 (18.7) | 460 (12.9) |  |

Data are expressed as proportions for categorical variables. BMI, body mass index.

#### Supplementary Table S3. Characteristics of studied population at baseline by total sleep duration

| **Variables** | **Total sleep duration (hours)** | | | | | ***P* value** |
| --- | --- | --- | --- | --- | --- | --- |
|  | **<6** | **6-<7** | **7-9** | **>9-10** | **>10** |  |
|  | **N=1846** | **N=1758** | **N=4945** | **N=704** | **N=320** |  |
| Male, n(%) | 765 (41.5) | 820 (46.6) | 2463 (49.8) | 360 (51.1) | 150 (46.9) | <0.001 |
| Age, n(%) |  |  |  |  |  | <0.001 |
| <50 | 375 (20.3) | 452 (25.7) | 1497 (30.3) | 197 (28.0) | 90 (28.1) |  |
| <60 | 665 (36.0) | 682 (38.8) | 1791 (36.2) | 237 (33.7) | 108 (33.8) |  |
| <70 | 590 (32.0) | 475 (27.0) | 1234 (25.0) | 183 (26.0) | 87 (27.1) |  |
| ≥70 | 216 (11.7) | 149 (8.5) | 423 (8.6) | 87 (12.4) | 35 (10.9) |  |
| BMI category (n, %) |  |  |  |  |  | <0.001 |
| <18.5 | 128 (6.9) | 88 (5.0) | 208 (4.2) | 29 (4.1) | 14 (4.4) |  |
| <24 | 1031 (55.9) | 887 (50.5) | 2492 (50.4) | 370 (52.6) | 166 (51.9) |  |
| <28 | 503 (27.3) | 564 (32.1) | 1588 (32.1) | 223 (31.7) | 90 (28.1) |  |
| ≥28 | 184 (10.0) | 219 (12.5) | 657 (13.3) | 82 (11.7) | 50 (15.6) |  |
| Rural residents, n(%) | 1180 (63.9) | 1026 (58.4) | 3020 (61.1) | 468 (66.5) | 222 (69.4) | <0.001 |
| Married, n(%) | 1512 (81.9) | 1510 (85.9) | 4301 (87.0) | 616 (87.5) | 257 (80.3) | <0.001 |
| Education level, n(%) |  |  |  |  |  | <0.001 |
| Below primary school | 140 (7.6) | 249 (14.2) | 716 (14.5) | 70 (9.9) | 23 (7.2) |  |
| Primary school | 360 (19.5) | 426 (24.2) | 1227 (24.8) | 177 (25.1) | 65 (20.3) |  |
| Middle school | 425 (23.0) | 423 (24.1) | 1190 (24.1) | 163 (23.2) | 85 (26.6) |  |
| High school or above | 921 (49.9) | 660 (37.5) | 1812 (36.6) | 294 (41.8) | 147 (45.9) |  |
| Ever smoking, n(%) | 678 (36.7) | 666 (37.9) | 1960 (39.6) | 293 (41.6) | 131 (40.9) | 0.081 |
| Current drinking, n(%) | 572 (31.0) | 616 (35.0) | 1792 (36.2) | 256 (36.4) | 107 (33.4) | 0.002 |
| Social activity, n(%) |  |  |  |  |  | <0.001 |
| 0 | 921 (49.9) | 827 (47.0) | 2194 (44.4) | 334 (47.4) | 146 (45.6) |  |
| 1 | 799 (43.3) | 774 (44.0) | 2245 (45.4) | 315 (44.7) | 147 (45.9) |  |
| 2 or more | 126 (6.8) | 157 (8.9) | 506 (10.2) | 55 (7.8) | 27 (8.4) |  |
| Hypertension, n(%) | 438 (23.7) | 360 (20.5) | 1008 (20.4) | 153 (21.7) | 85 (26.6) | 0.005 |
| Diabetes, n(%) | 111 (6.0) | 97 (5.5) | 262 (5.3) | 38 (5.4) | 16 (5.0) | 0.829 |
| Stroke, n(%) | 38 (2.1) | 32 (1.8) | 62 (1.3) | 11 (1.6) | 1 (0.3) | 0.037 |
| Heart disease, n(%) | 223 (12.1) | 171 (9.7) | 434 (8.8) | 48 (6.8) | 28 (8.8) | <0.001 |
| Psychiatric disease, n(%) | 30 (1.6) | 19 (1.1) | 38 (0.8) | 2 (0.3) | 3 (0.9) | 0.007 |
| Memory-related disease, n(%) | 19 (1.0) | 12 (0.7) | 33 (0.7) | 11 (1.6) | 0 (0.0) | 0.032 |
| Dyslipidemia, n(%) | 173 (9.4) | 160 (9.1) | 423 (8.6) | 57 (8.1) | 35 (10.9) | 0.475 |
| Cancer, n(%) | 22 (1.2) | 13 (0.7) | 35 (0.7) | 4 (0.6) | 2 (0.6) | 0.307 |
| Chronic lung disease, n(%) | 209 (11.3) | 126 (7.2) | 372 (7.5) | 55 (7.8) | 24 (7.5) | <0.001 |
| Sleep quality, n(%) |  |  |  |  |  | <0.001 |
| Good | 404 (7.9) | 758 (43.1) | 3197 (64.7) | 511 (72.6) | 245 (76.6) |  |
| Fair | 687 (37.2) | 703 (40.0) | 1350 (20.6) | 145 (20.6) | 53 (16.6) |  |
| Bad | 755 (49.7) | 297 (17.9) | 398 (8.1) | 48 (6.8) | 22 (6.9) |  |

Data are expressed as proportions for categorical variables. BMI, body mass index.

**Supplementary Table S4 Characteristics of the study population and participants for analysis**

| **Variables** | **Study population** | **Participants included in study** |
| --- | --- | --- |
|  | **N=11215** | **N=9573** |
| Male, n(%) | 5381 (48.0) | 4558 (47.6) |
| Age, n(%) |  |  |
| <50 | 2802 (25.0) | 2611 (27.3) |
| <60 | 3817 (36.8) | 3483 (36.4) |
| <70 | 2750 (26.5) | 2569 (26.8) |
| ≥70 | 991 (9.6) | 910 (9.5) |
| BMI category, n(%) |  |  |
| <18.5 | 531 (4.7) | 467 (4.9) |
| <24 | 5614 (50.8) | 4946 (51.7) |
| <28 | 3508 (31.8) | 2968 (31.0) |
| ≥28 | 1395 (12.6) | 1192 (12.5) |
| Rural residents, n(%) | 6857 (61.1) | 5916 (61.8) |
| Married, n(%) | 9629 (85.9) | 8196 (85.6) |
| Education level, n(%) |  |  |
| Below primary school | 1296 (12.6) | 1198 (12.5) |
| Primary school | 2401 (23.4) | 2255 (23.6) |
| Middle school | 2431 (23.7) | 2286 (23.9) |
| High school or above | 4148 (40.4) | 3834 (40.1) |
| Ever smoking, n(%) | 4047 (37.4) | 3728 (38.9) |
| Current drinking, n(%) | 3979 (35.5) | 3343 (34.9) |
| Social activity, n(%) |  |  |
| 0 | 5081 (45.3) | 4422 (46.2) |
| 1 | 5018 (44.7) | 4280 (44.7) |
| 2 or more | 1116 (10.0) | 871 (9.1) |
| Hypertension, n(%) | 2175 (21.6) | 2044 (21.4) |
| Diabetes, n(%) | 544 (5.4) | 524 (5.5) |
| Stroke, n(%) | 159 (1.6) | 144 (1.5) |
| Heart disease, n(%) | 966 (9.6) | 904 (9.4) |
| Psychiatric disease, n(%) | 100 (1.0) | 92 (1.0) |
| Memory-related disease, n(%) | 86 (0.9) | 75 (0.8) |
| Dyslipidemia, n(%) | 890 (9.0) | 848 (8.9) |
| Cancer, n(%) | 88 (0.9) | 76 (0.8) |
| Chronic lung disease, n(%) | 843 (8.4) | 786 (8.2) |
| Sleep quality, n(%) |  |  |
| Good | 5929 (53.1) | 5115 (53.4) |
| Fair | 3442 (30.9) | 2938 (30.7) |
| Bad | 1787 (16.0) | 1520 (15.9) |

#### Supplementary Table S5. Associations between total sleep duration and hearing loss

| **Total sleep duration (hours)** | **Case (%)** | **Crude incidence (1000 person-year)** | **Crude HR (95%CI)** | **Adjusted HR ^a^ (95%CI)** |
| --- | --- | --- | --- | --- |
| <6 | 297 (16.1) | 25.63 | 1.66 (1.44, 1.92) | 1.27 (1.08, 1.49) |
| 6-<7 | 186 (10.6) | 16.61 | 1.08 (0.91, 1.28) | 1.02 (0.86, 1.21) |
| 7-9 | 487 (9.9) | 15.39 | 1(ref) | 1(ref) |
| <9-10 | 69 (9.8) | 15.61 | 0.99 (0.77, 1.28) | 0.94 (0.73, 1.21) |
| >10 | 34 (10.6) | 17.64 | 1.08 (0.76, 1.53) | 1.00 (0.71, 1.42) |

^a^. Adjusted for gender, age (categorical), marital status, education level, area of residence, smoking status, drinking status, BMI, social activities, baseline chronic condition status and sleep quality.

#### Supplementary Table S6. Sensitivity analysis excluded people with BMI ≥30 kg/m^2^ (n= 9026)

| **Variables** | **Case (%)** | **Crude incidence (1000 person-year)** | **Crude HR (95%CI)** | **Adjusted HR ^a^ (95%CI)** |
| --- | --- | --- | --- | --- |
| **Nocturnal sleep duration (hours)** |  |  |  |  |
| <5 | 198 (18.2) | 30.11 | 2.02 (1.70, 2.40) | 1.64 (1.37, 1.95) |
| 5-<6 | 147 (12.0) | 18.45 | 1.24 (1.02, 1.51) | 1.10 (0.91, 1.34) |
| 6-<7 | 239 (10.9) | 17.32 | 1.18 (1.00, 1.39) | 1.17 (1.00, 1.38) |
| 7-<9 | 404 (9.4) | 14.91 | 1(ref) | 1(ref) |
| ≥9 | 85 (11.1) | 17.23 | 1.15 (0.91, 1.46) | 1.04 (0.81, 1.32) |
| **Midday napping duration (minutes)** |  |  |  |  |
| ≤5 | 581 (12.1) | 18.87 | 1(ref) | 1(ref) |
| 5-≤30 | 80 (9.2) | 15.00 | 0.77 (0.61, 0.97) | 0.81 (0.64, 1.03) |
| >30 | 365 (35.6) | 17.10 | 0.94 (0.83, 1.08) | 0.96 (0.84, 1.10) |

^a^. Adjusted for gender, age (categorical), marital status, education level, area of residence, smoking status, drinking status, BMI, social activities, baseline chronic condition status and sleep quality.

#### Supplementary Table S7. Sensitivity analysis reclassified nocturnal sleep duration into three groups

| **Variables** | **Case (%)** | **Crude incidence (1000 person-year)** | **Crude HR (95%CI)** | **Adjusted HR ^a^ (95%CI)** |
| --- | --- | --- | --- | --- |
| **Nocturnal sleep duration (hours)** |  |  |  |  |
| <7 | 584 (13.0) | 20.48 | 1.40 (1.23, 1.60) | 1.18 (1.02, 1.35) |
| 7-<9 | 404 (9.4) | 14.75 | 1(ref) | 1(ref) |
| ≥9 | 85 (11.1) | 17.26 | 1.15 (0.91, 1.46) | 1.05 (0.83, 1.34) |
| **Midday napping duration (minutes)** |  |  |  |  |
| ≤5 | 581 (12.1) | 18.87 | 1(ref) | 1(ref) |
| ≤30 | 80 (9.2) | 15.00 | 0.76 (0.60, 0.96) | 0.81 (0.64, 1.02) |
| >30 | 365 (35.6) | 17.10 | 0.93 (0.81, 1.06) | 0.95 (0.83, 1.09) |

^a^. Adjusted for gender, age (categorical), marital status, education level, area of residence, smoking status, drinking status, BMI, social activities, baseline chronic condition status and sleep quality.

#### Supplementary Table S8. Sensitivity analysis reset 7-8 hours/night as the reference group

| **Variables** | **Case (%)** | **Crude incidence (1000 person-year)** | **Crude HR (95%CI)** | **Adjusted HR ^a^ (95%CI)** |
| --- | --- | --- | --- | --- |
| **Nocturnal sleep duration (hours)** |  |  |  |  |
| <5 | 198 (18.2) | 29.22 | 2.00 (1.68, 2.37) | 1.46 (1.20, 1.76) |
| 5-<6 | 147 (12.0) | 18.83 | 1.29 (1.07, 1.56) | 1.05 (0.87, 1.28) |
| 6-<7 | 239 (10.9) | 17.20 | 1.19 (1.01, 1.39) | 1.13 (0.96, 1.33) |
| 7-8 | 402 (9.4) | 14.72 | 1(ref) | 1(ref) |
| >9 | 87 (11.1) | 17.33 | 1.18 (0.94, 1.49) | 1.08 (0.85, 1.36) |
| **Midday napping duration (minutes)** |  |  |  |  |
| ≤5 | 581 (12.1) | 18.64 | 1(ref) | 1(ref) |
| ≤30 | 80 (9.2) | 14.50 | 0.76 (0.60, 0.95) | 0.80 (0.63, 1.00) |
| >30 | 365 (35.6) | 17.08 | 0.95 (0.84, 1.09) | 0.97 (0.85, 1.11) |

^a^. Adjusted for gender, age (categorical), marital status, education level, area of residence, smoking status, drinking status, BMI, social activities, baseline chronic condition status and sleep quality.

**Supplementary Table S9 Evaluation of missing data using complete data and multiple imputation methods.**

| **Variables** | | **Adjusted HR ^a^ (95%CI)** | |
| --- | --- | --- | --- |
|  |  | **Complete data** | **Multiple imputation** |
| **Nocturnal sleep duration (hours)** | |  |  |
| <5 | | 1.45 (1.20, 1.75) | 1.45 (1.21, 1.73) |
| 5-<6 | | 1.04 (0.86, 1.28) | 1.08 (0.90, 1.29) |
| 6-<7 | | 1.12 (0.96, 1.32) | 1.11 (0.95, 1.29) |
| 7-<9 | | 1(ref) | 1(ref) |
| ≥9 | | 1.07 (0.84, 1.35) | 1.13 (0.91, 1.41) |
| **Midday napping duration (minutes)** | |  |  |
| ≤5 | | 1(ref) | 1(ref) |
| 5-≤30 | | 0.80 (0.63, 1.00) | 0.81 (0.65, 1.00) |
| >30 | | 0.97 (0.82, 1.11) | 0.94 (0.83, 1.06) |
| **Nocturnal sleep duration (hours)** | **Midday napping duration (minutes)** |  |  |
| <7 | ≤5 | 1.27 (1.06, 1.52) | 1.24 (1.05, 1.46) |
| 7-<9 | ≤5 | 1(ref) | 1(ref) |
| ≥9 | ≤5 | 1.29 (0.95, 1.74) | 1.26 (0.94, 1.68) |
| <7 | 5-≤30 | 0.90 (0.65, 1.25) | 0.88 (0.65, 1.20) |
| 7-<9 | 5-≤30 | 1.03 (0.72, 1.49) | 0.97 (0.73, 1.38) |
| ≥9 | 5-≤30 | 0.53 (0.17, 1.66) | 0.65 (0.24, 1.75) |
| <7 | >30 | 1.19 (0.97, 1.46) | 1.14 (0.94, 1.38) |
| 7-<9 | >30 | 1.06 (0.87, 1.31) | 1.03 (0.85, 1.25) |
| ≥9 | >30 | 0.94 (0.63, 1.40) | 1.01 (0.70, 1.45) |

a. Adjusted for gender, age (categorical), marital status, education level, area of residence, smoking status, drinking status, BMI, social activities, baseline chronic condition status and sleep quality.
